# Stepwise evolution of tigecycline resistance in *Enterococcus hirae* through early IS*1216E*-mediated *tet*(L) amplification and subsequent *rpsJ* mutations

**DOI:** 10.1186/s13567-026-01853-4

**Published:** 2026-10-10

**Authors:** Yanbin Dong, Zhongyi Fang, Zhengyu Wei, Kun He, Guimei Zhang, Shuaijia Yang, RuiTing Geng, Yushan Pan, Dandan He, Xiaoyuan Ma, Li Yuan

**Affiliations:** 1 College of Veterinary Medicine, Henan Agricultural University, Zhengzhou, 450046 China; 2 Henan Institute of Technical Research on Agricultural Product, Zhengzhou, 450008 China; 3 Key Laboratory of Quality and Safety Control of Poultry Products (Zhengzhou), Ministry of Agriculture and Rural Affairs, Zhengzhou, 450046 China; 4 Ministry of Education Key Laboratory for Animal Pathogens and Biosafety, Zhengzhou, 450046 China

**Keywords:** *E. hirae*, tigecycline resistance, translocatable units, *tet*(L), *rpsJ*

## Abstract

*Enterococcus * spp. from food animals represent important reservoirs of antimicrobial resistance; however, the mechanisms underlying tigecycline resistance remain poorly understood. In this study, we investigated the genetic basis and evolutionary dynamics of tigecycline resistance in *Enterococcus hirae*. Among 118 *Enterococcus* isolates recovered from 150 swine-derived samples, *E. hirae* was the predominant species (68/118, 57.63%). Tigecycline resistance was identified in ten isolates (10/118, 8.47%), all belonging to a single *E. hirae* clone. Whole-genome sequencing of a representative isolate, EG2361, revealed three plasmids, among which pEG2361-2 harbored an IS*1216E*-associated multidrug resistance region capable of generating two circular translocatable units (TUs). TU1, carrying the promoter-variant *tet*(L) locus together with *tet*(M) and *poxtA*, underwent tandem amplification, leading to plasmid structural heterogeneity. Functional analyses demonstrated that *tet*(L) reduced tigecycline susceptibility, whereas enhanced resistance mainly associated with variation in the upstream promoter region rather than Tet(L) amino acid substitutions. Experimental evolution revealed a dynamic resistance trajectory. The initial increase in tigecycline resistance was associated with TU1 amplification and marked upregulation of *tet*(L) expression. Subsequently, *tet*(L) expression declined, whereas tigecycline minimum inhibitory concentrations (MICs) remained elevated and stable. Recurrent *rpsJ* mutations emerged independently across evolved lineages and were associated with high-level resistance. Together, these findings support a stepwise evolutionary model in which insertion sequence (IS)*1216E*-mediated amplification of the promoter-variant *tet*(L) locus drives rapid early adaptation, followed by the emergence of chromosomal *rpsJ* mutations associated with stable high-level resistance. This study expands current understanding of tigecycline resistance evolution in enterococci and highlights the interplay among mobile genetic elements, transcriptional regulation, and chromosomal adaptation in shaping antimicrobial resistance.

**Supplementary Information:**

The online version contains supplementary material available at https://doi.org/10.1186/s13567-026-01853-4.

## Introduction

Tigecycline, a glycylcycline antibiotic structurally related to tetracyclines, inhibits bacterial protein synthesis by binding to the 30S ribosomal subunit. Owing to its broad-spectrum activity against multidrug-resistant (MDR) pathogens, tigecycline has become an important therapeutic option for severe infections caused by vancomycin-resistant enterococci (VRE) [1] and other MDR Gram-positive bacteria [2]. However, increasing reports of reduced tigecycline susceptibility threaten its clinical utility [3] and highlight the need to better understand the underlying resistance mechanisms.

Although tigecycline is not approved for veterinary use in China [4], tetracyclines such as doxycycline and chlortetracycline remain widely used in livestock production. Previous reports have demonstrated that the extensive use of tetracyclines in both human and animal settings can co-select resistance determinants that confer reduced susceptibility to tigecycline [5–7], thereby facilitating the emergence and persistence of tigecycline-resistant bacteria across the One Health ecosystem. In this context, food-producing animals may serve as important reservoirs of such resistance determinants, with potential public health relevance.

Across bacterial species, tigecycline resistance is generally associated with three major mechanisms: (i) target-site alterations, including mutations in ribosomal protein S10 encoded by *rpsJ* [8]; (ii) increased drug efflux mediated by multidrug transporters [9, 10]; and (iii) acquisition of transferable resistance genes such as *tet*(X) [11] and *tmexCD-toprJ* [12]. Although these mechanisms have been extensively studied in Gram-negative bacteria [13, 14], their contribution to tigecycline resistance evolution in enterococci remains incompletely understood.

Several recent studies have suggested that classical tetracycline resistance determinants may also contribute to reduced tigecycline susceptibility in some Gram-positive bacteria. For example, variants of *tet*(L) in *Staphylococcus aureus* [15] and *tet*(M) in *Streptococcus suis* [16] have been associated with elevated tigecycline minimum inhibitory concentrations (MICs). In Gram-positive bacteria, these genes are frequently associated with mobile genetic elements, particularly insertion sequence (IS)*1216*/IS*1216E*-like elements, which can mediate gene mobilization and structural rearrangements through recombination. However, whether IS*1216E*-associated structural plasticity of resistance regions contributes to the adaptive evolution of tigecycline resistance in enterococci remains largely unexplored.

Enterococci are common commensals of the gastrointestinal tract of humans and animals [17] but are also important opportunistic pathogens capable of causing bacteremia, urinary tract infections, endocarditis, and wound infections [18, 19]. Their ecological adaptability and remarkable capacity to acquire antimicrobial resistance make them key organisms within the One Health framework. Among them, *E. hirae* is frequently recovered from animals and is increasingly recognized as a reservoir of antimicrobial resistance determinants [20, 21], yet the evolutionary mechanisms of tigecycline resistance in this species remain largely unexplored.

In this study, we investigated the antimicrobial resistance profiles of 118 *Enterococcus* isolates recovered from a swine farm in Henan province, China, with particular focus on tigecycline-resistant *E. hirae*. We further characterized the genetic basis and adaptive evolution of tigecycline resistance in *E. hirae*, aiming to provide new insights into the emergence and evolution of tigecycline resistance in animal-associated enterococci.

## Materials and methods

### Bacterial isolates

A total of 150 samples, including 75 rectal swabs and 75 pharyngeal swabs, were collected from apparently healthy pigs at a swine farm in Henan Province, China, in December 2023 as part of an antimicrobial resistance surveillance program. All samples were pre-enriched in 5 mL Brain Heart Infusion (BHI) broth and incubated at 37 °C for 12 h. Subsequently, 10 μL of each enriched culture was streaked in duplicate onto *Enterococcus* selective agar plates (Hopebio, Qingdao, China) and incubated at 37 °C for 24 h. Presumptive *Enterococcus* colonies were selected based on colony morphology, purified, and identified using the VITEK 2 Compact system (bioMérieux, Marcy-l’Étoile, France), with species assignment further confirmed by 16S ribosomal RNA (rRNA) gene sequencing. One representative isolate from each distinct colony morphotype per sample was retained for subsequent analysis.

### Antimicrobial susceptibility testing

Antimicrobial susceptibility of all isolates was determined using the broth microdilution method. The antimicrobial agents tested were linezolid, vancomycin, tigecycline, florfenicol, doxycycline, enrofloxacin, lincomycin (OriLeaf, Shanghai, China), and tylosin (Solarbio, Beijing, China). MICs of linezolid (≥ 8 mg/L), vancomycin (≥ 32 mg/L), and doxycycline (≥ 16 mg/L) were interpreted according to the Clinical and Laboratory Standards Institute (CLSI) VET01S criteria (CLSI, 2025). Tigecycline resistance was defined as a MIC of > 0.5 mg/L according to the European Committee on Antimicrobial Susceptibility Testing (EUCAST) Clinical Breakpoint Tables, version 15.0.

As no directly applicable CLSI or EUCAST clinical breakpoints were available for florfenicol, enrofloxacin, lincomycin, or tylosin in *Enterococcus * spp., interpretive thresholds previously applied in published studies were used. The resistance thresholds for florfenicol (≥ 32 mg/L) and enrofloxacin (≥ 2 mg/L) were adopted from Bzdil et al. [22], whereas those for lincomycin (≥ 8 mg/L) and tylosin (≥ 32 mg/L) were adopted from Cho et al. [23]. *Enterococcus faecalis* ATCC 29212 was included as the quality-control strain. All MIC determinations were performed in triplicate.

### Pulsed-field gel electrophoresis

Tigecycline-resistant *Enterococcus* isolates were subjected to pulsed-field gel electrophoresis (PFGE) to assess clonal relatedness, as previously described [24]. Briefly, bacterial cell suspensions were embedded in low-melting-point agarose plugs (Bio-Rad, Hercules, CA, USA), lysed, and the genomic DNA was digested with the restriction enzyme SmaI (TaKaRa, Japan) prior to PFGE analysis. DNA fragments were separated using a CHEF Mapper XA system (Bio-Rad, USA) under previously described running conditions. *Salmonella enterica* serotype Braenderup H9812 was used as the molecular size standard. Gels were stained, visualized using a Gel Doc imaging system (Bio-Rad, USA), and analyzed with BioNumerics software version 6.6 (Applied Maths, Sint-Martens-Latem, Belgium).

### Whole-genome sequencing and bioinformatics analysis

*E. hirae* EG2361 was selected for whole-genome sequencing (WGS) based on its phenotypic characteristics. Genomic DNA was extracted using the E.Z.N.A.® Bacterial DNA Kit (Omega Bio-tek, USA) according to the manufacturer’s instructions. Illumina sequencing libraries were prepared using the TruSeq™ DNA Sample Prep Kit (Illumina, USA) following the manufacturer’s protocol. Short-read sequencing (2 × 150 bp) was performed on the Illumina NovaSeq platform, and long-read sequencing was conducted using the Oxford Nanopore Technologies MinION platform. Raw Illumina reads were quality-filtered and adapter-trimmed using AdapterRemoval [25]. Nanopore long reads were de novo assembled using Flye [26]. The resulting assemblies were subsequently polished with Illumina short reads through five iterative rounds using Pilon v1.23 [27]. Plasmid sequences were functionally annotated using the Rapid Annotation using Subsystem Technology (RAST) server [28]. Genes potentially associated with conjugative transfer were identified based on the annotation results, followed by manual inspection of their genomic organization and surrounding plasmid regions. Antibiotic resistance genes (ARGs) and plasmid replicon types were identified using ResFinder 4.6 [29] and PlasmidFinder [30], respectively. IS elements were identified using ISfinder [31]. The genetic context of *tet*(L) homologs was visualized using Easyfig [32].

### Detection of circular translocatable units (TUs)

The presence of IS*1216E*-mediated TUs was investigated by inverse polymerase chain reaction (PCR) using outward-facing primers targeting predicted recombination junctions. PCR products were validated by Sanger sequencing. Primer sequences are listed in Additional file 1.

### S1-PFGE and southern blot hybridization of *E. hirae* EG2361

To investigate plasmid size variation in *E. hirae* EG2361, ten individual colonies were randomly selected (designated S1–S10), cultured overnight in BHI broth at 37 °C, and subjected to S1 nuclease pulsed-field gel electrophoresis (S1-PFGE). Briefly, genomic DNA was prepared in agarose plugs and digested with S1 nuclease. Linearized DNA fragments were separated using a CHEF-DR III system (Bio-Rad, Hercules, CA, USA) under the following conditions: 6 V/cm, included angle of 120°, run time of 18 h, initial switch time of 2.16 s, and final switch time of 63.8 s at 14 °C in 0.5 × Tris–borate-EDTA (TBE) buffer. *XbaI*-digested DNA of *Salmonella enterica* serotype Braenderup H9812 was used as the molecular size standard.

For southern blot hybridization, DNA fragments were transferred to nylon membranes and hybridized with digoxigenin (DIG)-labeled *tet*(L) and *poxtA* probes prepared using a DIG DNA Labeling and Detection Kit (Roche Diagnostics, Basel, Switzerland), according to the manufacturer’s instructions.

### Sequence analysis and functional characterization of the *tet*(L) variant and its promoter

The *tet*(L) variant identified in *E. hirae* EG2361 was characterized by amino acid sequence alignment with Tet(L) from the tigecycline-susceptible isolate EG2363 and the reference sequence encoded by plasmid pG38 (GenBank accession no. RCDF01000030) using ClustalW. Amino acid sequence alignments were visualized using the ESPript web server. The open reading frame of *tet*(L) and its upstream promoter region were predicted using BPROM. Four DNA fragments were amplified for functional analysis: (i) the wild-type *tet*(L) [designated *tet*(L)], (ii) the variant *tet*(L) [designated *tet*(L)v], (iii) wild-type *tet*(L) with a 250-bp upstream region containing the predicted promoter [designated *tet*(L)_250], and (iv) *tet*(L)v with the corresponding 250-bp upstream region [designated *tet*(L)v_250]. Primer sequences are listed in Additional file 1, whereas the amino acid substitutions and promoter regions are presented in Additional file 2.

PCR products were purified using a gel extraction kit (Omega Bio-tek, Norcross, GA, USA) and ligated into the pMD19-T vector (TaKaRa Bio Inc., Japan). The ligation mixtures were transformed into chemically competent *Escherichia coli* DH5α cells. X-Gal and IPTG were spread on BHI agar plates containing ampicillin (100 mg/L), and transformants were then inoculated and incubated at 37 °C for 12 h. White colonies were selected and further confirmed by colony PCR and sequencing. Antimicrobial susceptibility of recombinant strains to tetracycline, doxycycline, minocycline, and tigecycline was determined by broth microdilution as described above.

### Experimental evolution of tigecycline resistance in *E. hirae* EG2361

A single colony of *E. hirae* EG2361 carrying pEG2361-2 with a single TU1 copy and no TU1 tandem amplification was selected as the parental strain and cultured in 5 mL of BHI broth. The culture was diluted 1:100 to establish three tigecycline-exposed lineages (Ta, Tb, and Tc) and three drug-free control lineages (Ca, Cb, and Cc). For experimental evolution, tigecycline-exposed populations were initially cultured in BHI broth containing a subinhibitory concentration of tigecycline (0.5 mg/L), corresponding to approximately 50% growth inhibition, and incubated at 37 °C with shaking at 180 rpm for 12 h. After each passage, 50 μL of culture was transferred into fresh medium containing tigecycline at 0.5-fold, 1-fold, and 2-fold of the concentration used in the previous passage. Cell growth was monitored by measuring optical density at 600 nm (OD_600_), and only the population grown at the highest tigecycline concentration with OD_600_ > 0.8 was selected for the next passage.

**Figure 1:**
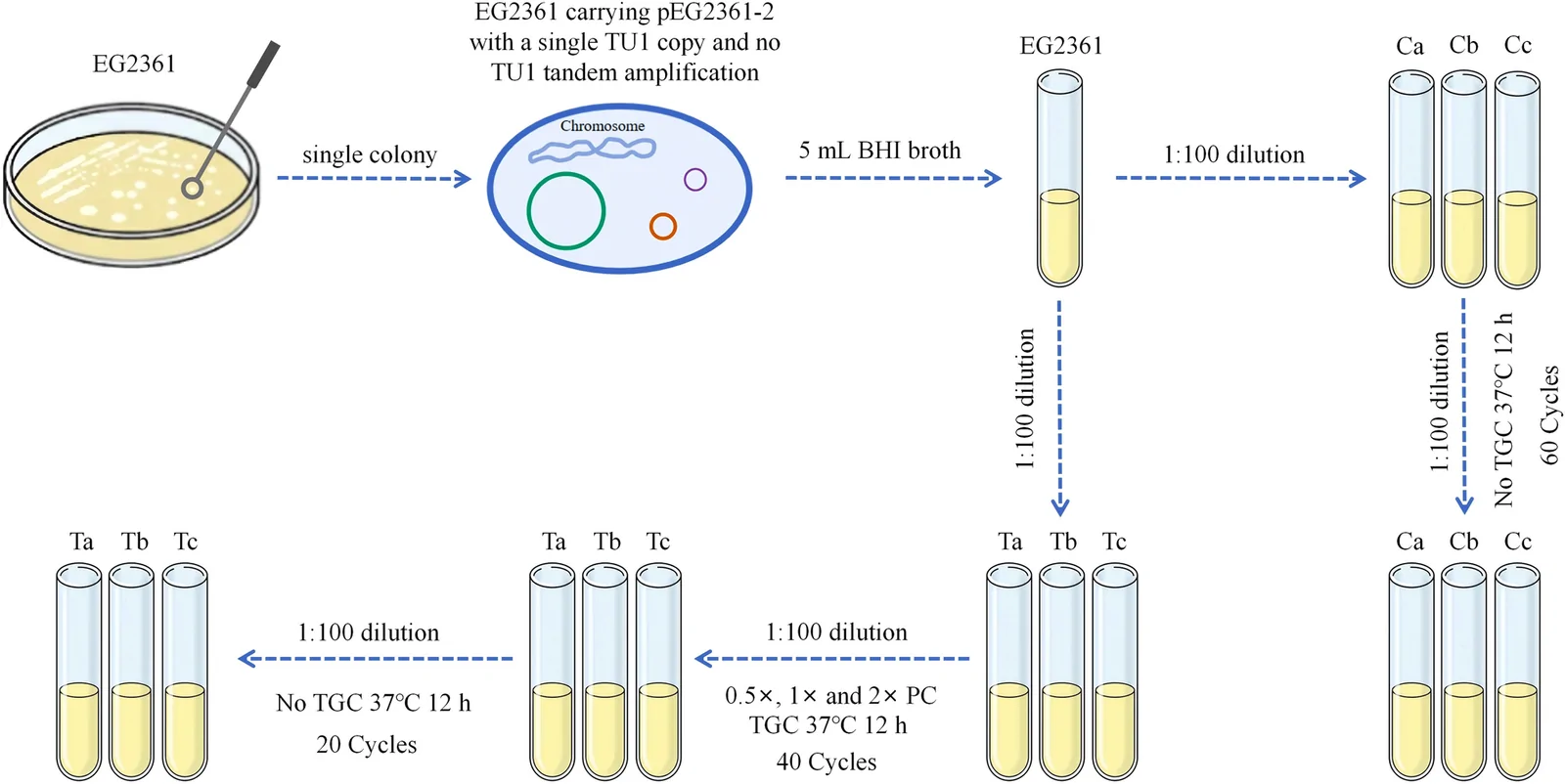
**Schematic of experimental evolution in** ***E. hirae*** **EG2361**. A single colony of EG2361 carrying pEG2361-2 with a single TU1 copy and no TU1 tandem amplification was used to establish three tigecycline-treated lineages (Ta, Tb, and Tc) and three untreated control lineages (Ca, Cb, and Cc). The treated lineages were serially passaged every 12 h at 37 °C for 40 cycles under tigecycline selection at 0.5×, 1×, or 2× the previous concentration (PC), followed by 20 additional cycles without antibiotic. In parallel, control lineages were passaged for 60 cycles in the absence of tigecycline. TGC, tigecycline; PC, previous concentration. The green, orange, and purple circles indicate pEG2361-1, pEG2361-2, and pEG2361-3, respectively.

The adaptive evolution experiment was continued for 40 serial passages (20 days). Subsequently, evolved populations were transferred to drug-free BHI broth and passaged for an additional 20 cycles (10 days) to assess resistance stability. In parallel, control lineages were serially passaged every 12 h in antibiotic-free BHI broth for a total of 60 passages (30 days) (Figure 1). Tigecycline MICs were determined at designated timepoints by the broth microdilution method as described above.

### Real-time relative quantitative PCR analysis of ***tet***(L), ***tet***(M), and ***poxtA*** expression levels

Evolved lineages were collected at different timepoints (0, 2.5, 7.5, 12.5, 17.5, and 20 days) and drug-free subcultured populations (25 and 30 days) were cultured in BHI broth at 37 °C. Overnight cultures were diluted 1:100 in fresh medium and grown to an OD_600_ of 0.8. Total RNA was extracted using the MiniBEST Universal RNA Extraction Kit (TaKaRa Bio, Inc., Shiga, Japan) according to the manufacturer’s instructions. RNA concentration and purity were determined using a NanoDrop 1000 spectrophotometer (Thermo Scientific, Denmark). First-strand complementary DNA (cDNA) was synthesized using the PrimeScript RT reagent kit with gDNA Eraser (TaKaRa Bio, Inc.). RT-qPCR was performed at an annealing temperature of 60 °C as previously described [33]. The transcription levels of *tet*(L), *tet*(M), and *poxtA* were quantified using the 2^−ΔΔCt^ method with the day 0 parental strain as the calibrator. The 16S rRNA gene was used as the internal reference gene. Primer sequences are listed in Additional file 1. All experiments were performed with three independent biological replicates.

### Detection of *rpsJ* mutations during experimental evolution

The *rpsJ* gene was amplified and sequenced from tigecycline-exposed lineages (Ta, Tb, and Tc) collected at 2.5, 7.5, 12.5, 17.5, and 20 days during experimental evolution, as well as from populations collected at 25 and 30 days during the subsequent drug-free passaging period. At each timepoint, four colonies were randomly selected from each lineage for PCR amplification and sequencing. Mutations in *rpsJ* were identified by comparison with the parental EG2361 sequence. Primer sequences used in this study are listed in Additional file 1.

### Statistical analysis

Statistical analyses were performed using GraphPad Prism version 10.0. Intergroup differences were assessed by an unpaired *t*-test (for two groups) or one-way analysis of variance (ANOVA) (for more than two groups). Statistical significance was defined as follows: *p < 0.05, **p < 0.01, ***p < 0.001, and ****p < 0.0001. Unless otherwise specified, all data are presented as the mean ± standard deviation (SD) from at least three separate experiments.

## Results

### Isolation rate and species distribution of *Enterococcus* spp. across sample sources

**Figure 2:**
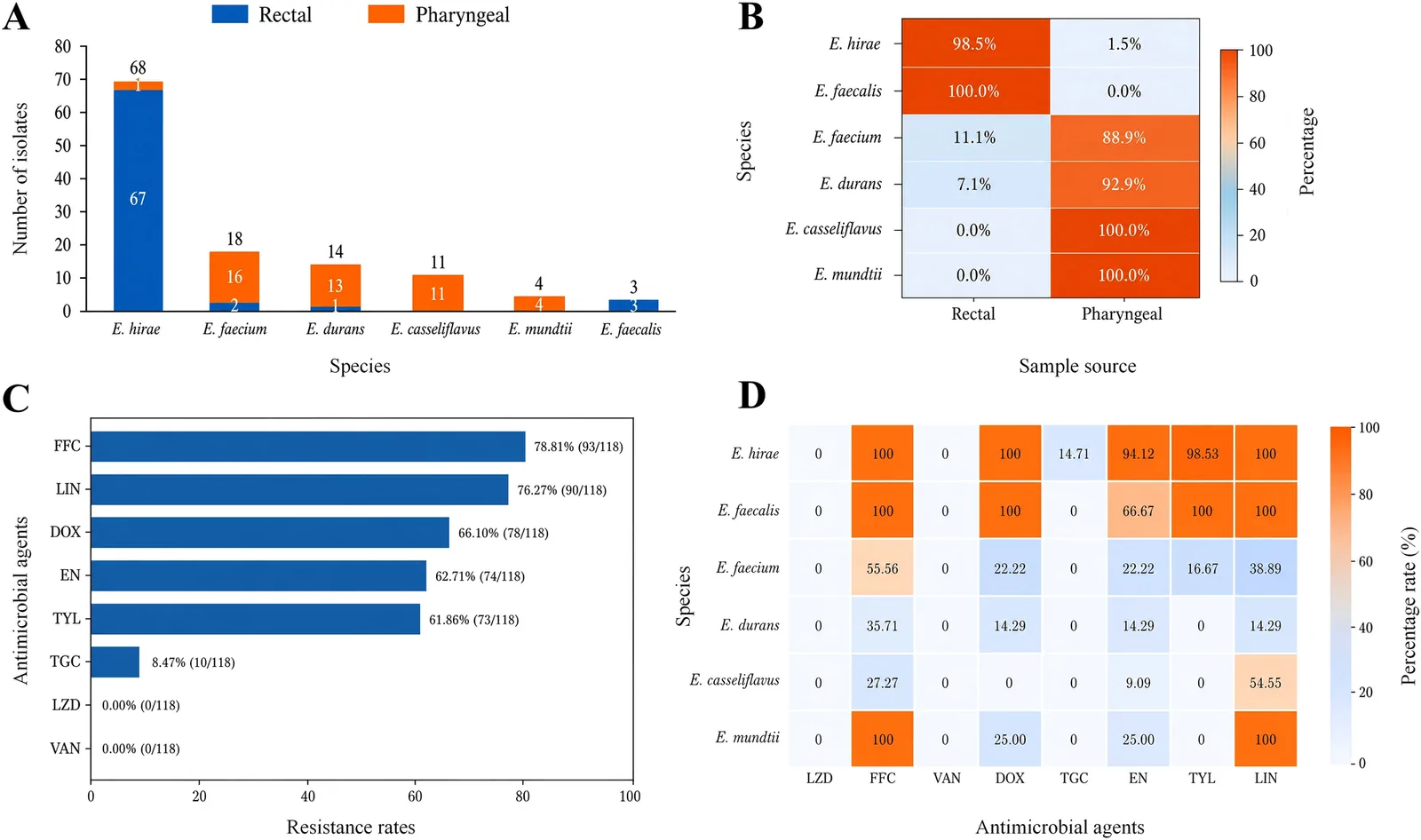
**Species distribution and antimicrobial resistance profiles of** ***Enterococcus*** **isolates.** **A** Distribution of *Enterococcus* species across different sample sources. Blue and orange bars represent rectal and pharyngeal isolates, respectively. **B** Heatmap showing the proportion of each species in rectal and pharyngeal samples. **C** Overall resistance rates of the 118 isolates to different antimicrobial agents; values indicate percentages and the number of resistant isolates. **D** Species-specific resistance rates to different antimicrobial agents. FFC, florfenicol; LIN, lincomycin; DOX, doxycycline; EN, enrofloxacin; TYL, tylosin; TGC, tigecycline; LZD, linezolid; VAN, vancomycin.

A total of 118 *Enterococcus* isolates were recovered from 150 swine-derived samples (75 rectal swabs and 75 pharyngeal swabs), corresponding to an overall isolation rate of 78.7%. Species identification showed that *E. hirae* was the predominant species (68/118, 57.63%), followed by *E. faecium* (18/118, 15.25%), *E. durans* (14/118, 11.86%), *E. casseliflavus* (11/118, 9.32%), *E. mundtii* (4/118, 3.39%), and *E. faecalis* (3/118, 2.54%) (Figure 2A, Additional file 3).

Clear differences in species distribution were observed between sample sources (Figure 2B). *E. hirae* was almost exclusively isolated from rectal swabs (67/68, 98.53%), and all *E. faecalis* isolates (3/3, 100%) were also derived from rectal samples. In contrast, *E. casseliflavus* (11/11, 100%) and *E. mundtii* (4/4, 100%) were exclusively recovered from pharyngeal swabs, while *E. durans* (13/14, 92.86%) and *E. faecium* (16/18, 88.89%) were predominantly associated with pharyngeal samples. These results indicate a strong species-specific preference for colonization at distinct anatomical sites.

### Antimicrobial resistance profiles of *Enterococcus* spp. with low but species-restricted tigecycline resistance

Antimicrobial susceptibility testing revealed generally high resistance rates among the isolates (Figure 2C). The highest resistance was observed for florfenicol (93/118, 78.81%) and lincomycin (90/118, 76.27%), followed by doxycycline (78/118, 66.10%), enrofloxacin (74/118, 62.71%), and tylosin (73/118, 61.86%). In contrast, resistance to tigecycline was relatively low (10/118, 8.47%), and all isolates remained fully susceptible to vancomycin and linezolid, indicating preserved activity of last-resort antibiotics.

Notably, tigecycline-resistant bacteria were all *E. hirae* (Figure 2D). Given the clinical importance of tigecycline and its retained activity against most isolates, these findings highlight tigecycline resistance in *E. hirae* as a key issue warranting further investigation. Accordingly, tigecycline-resistant *E. hirae* isolates were selected for subsequent genomic and functional analyses.

Species-level analysis revealed marked heterogeneity in resistance profiles (Figure 2D, Additional file 3). *E. faecalis* exhibited the most pronounced multidrug-resistant phenotype, with complete resistance to florfenicol, lincomycin, doxycycline, and tylosin, and high resistance to enrofloxacin (2/3, 66.67%). *E. hirae* also displayed broadly elevated resistance rates across multiple antibiotic classes, including florfenicol (68/68, 100%), doxycycline (68/68, 100%), enrofloxacin (64/68, 94.12%), tylosin (67/68, 98.53%), and lincomycin (68/68, 100%). In contrast, *E. faecium* showed moderate resistance levels, whereas *E. durans* and *E. casseliflavus* exhibited generally low resistance rates. *E. mundtii* displayed a distinct pattern, with high resistance to florfenicol and lincomycin but lower resistance to other antimicrobial agents.

### Genomic basis of tigecycline resistance in *E. hirae* EG2361

PFGE analysis of the ten tigecycline-resistant *E. hirae* isolates revealed identical banding patterns (Additional file 4A), indicating that these isolates have close relatedness. Based on this finding, isolate EG2361 was selected as a representative strain for WGS.

The genome of EG2361 consisted of a 2,920,071 bp chromosome (GC content, 36.89%) carrying a single aminoglycoside resistance gene, *aac(*6′*)-Iid*, together with three plasmids (Additional file 5). Plasmid pEG2361-1 (137,737 bp; GC content 35.53%) belonged to the RepA_N family (repUS15-type replicon) and carried multiple resistance genes conferring resistance to aminoglycosides, macrolides, and lincosamides. Plasmid pEG2361-2 (33,213 bp; GC content 35.75%) was an Inc18-type plasmid (rep2-type replicon) harboring *poxtA*, *tet*(L), *tet*(M), and *fexB*, associated with resistance to amphenicols, tetracyclines, and oxazolidinones. However, despite the presence of *poxtA*, the isolate remained susceptible to linezolid, possibly due to its limited contribution to resistance or insufficient expression under the tested conditions. Plasmid pEG2361-3 (23,624 bp; GC content 34.40%) was also an Inc18-type plasmid (repUS1-type replicon) carrying *erm*(B) and *dfrG*. Further analysis showed that none of the three plasmids contained a complete known conjugative transfer region; however, the possibility of horizontal transfer through uncharacterized mechanisms cannot be excluded.

**Figure 3:**
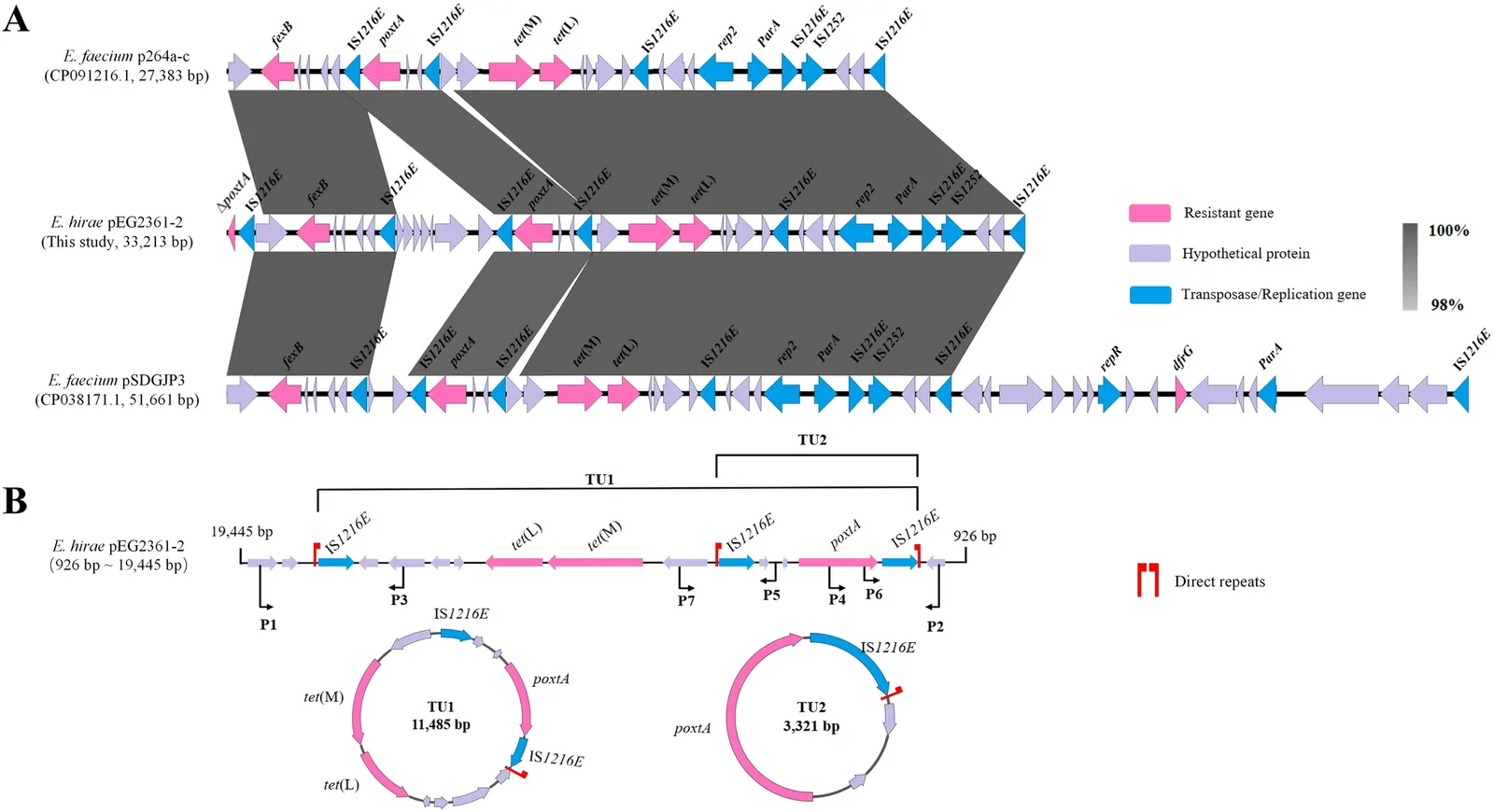
**Genetic organization of the IS*1216E*-associated resistance region in plasmid pEG2361-2.** **A** Comparison of the pEG2361-2 sequence from *E. hirae* EG2361 with homologous regions in *E. faecium* plasmids p264a-c (CP091216.1) and pSDGJP3 (CP038171.1). Arrows indicate genes and open reading frames (ORFs), and their direction represent transcriptional orientation. **B** Identification of circular TUs derived from plasmid pEG2361-2. TU1 and TU2 correspond to the *tet*(L)–*tet*(M)–IS*1216E*–*poxtA*–IS*1216E* and *poxtA*–IS*1216E* modules, respectively. P1–P7 indicate primer binding sites.

Of note, tetracycline resistance genes were exclusively located on pEG2361-2 and were absent from the chromosome and the other two plasmids, indicating that this plasmid represents the primary genetic determinant of tigecycline resistance in EG2361. Detailed analysis of pEG2361-2 showed that both the *tet*(L)–*tet*(M) region and the *poxtA* module were flanked by IS*1216E* elements in the same orientation (Figure 3A), forming typical IS*1216E*-resistance gene-IS*1216E* structures. To assess their ability to generate circular intermediates, inverse PCR was performed using primers targeting predicted recombination junctions. Multiple amplicons were obtained (Additional file 6), and Sanger sequencing confirmed the presence of two circular TUs: TU1 (~11.5 kb), carrying *tet*(L)–*tet*(M)–IS*1216E*–*poxtA*–IS*1216E*, and TU2 (~3.3 kb), consisting of the *poxtA*–IS*1216E* module (Figure 3B). In contrast, no amplification was obtained with the primer pair P3/P7, suggesting that the IS*1216E*–*tet*(L)-*tet*(M)–IS*1216E* structure had limited capacity to circularize under the tested conditions.

### Structural heterogeneity of plasmid pEG2361-2 driven by TU1 amplification

**Figure 4:**
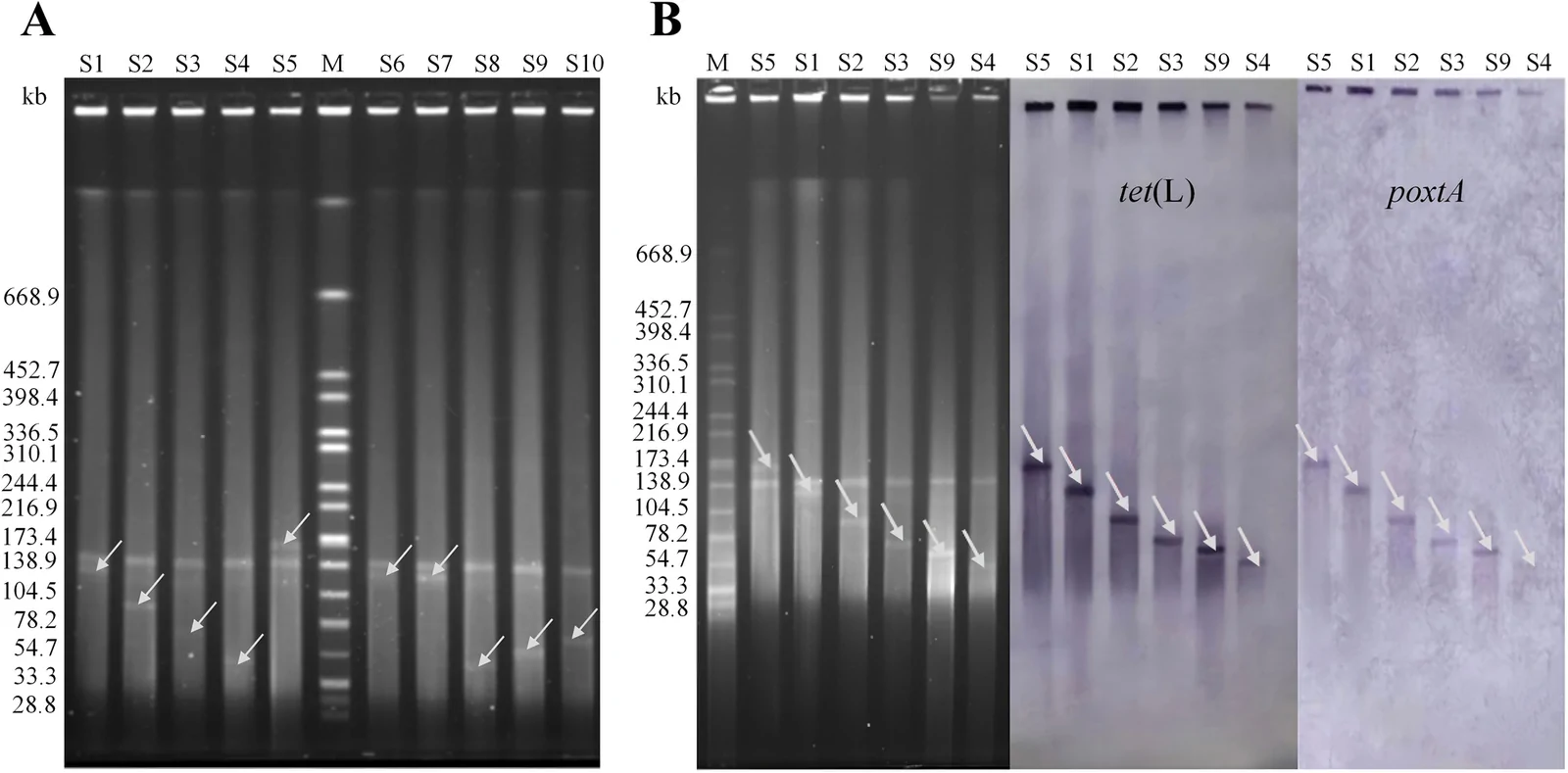
**S1-PFGE profiles and southern blot analysis of plasmids from ten individual colonies of** ***E. hirae*** **EG2361.** **A** S1-PFGE profiles (S1–S10). **B** Southern blot hybridization detecting *tet(L)* and *poxtA*. M, *Salmonella enterica* serotype Braenderup H9812.

To determine whether TU1 contributes to plasmid heterogeneity, ten individual colonies of *E. hirae* EG2361 were randomly selected and analyzed by S1-PFGE. All colonies consistently harbored a conserved plasmid (~138 kb), corresponding to pEG2361-1, together with a second plasmid exhibiting substantial size variation, ranging from ~45 kb to ~155 kb (Figure 4A). Based on these profiles, the colonies were classified into six distinct S1-PFGE types (Additional file 7), with size differences occurring in ~11 kb increments.

Southern blot analysis confirmed that both *tet*(L) and *poxtA* were located on the variable plasmid, confirming it as pEG2361-2 (Figure 4B). Notably, the observed ~11 kb size increments closely matched the length of TU1 (~11,485 bp), suggesting that tandem amplification of TU1 underlies the observed plasmid size variation.

**Figure 5:**
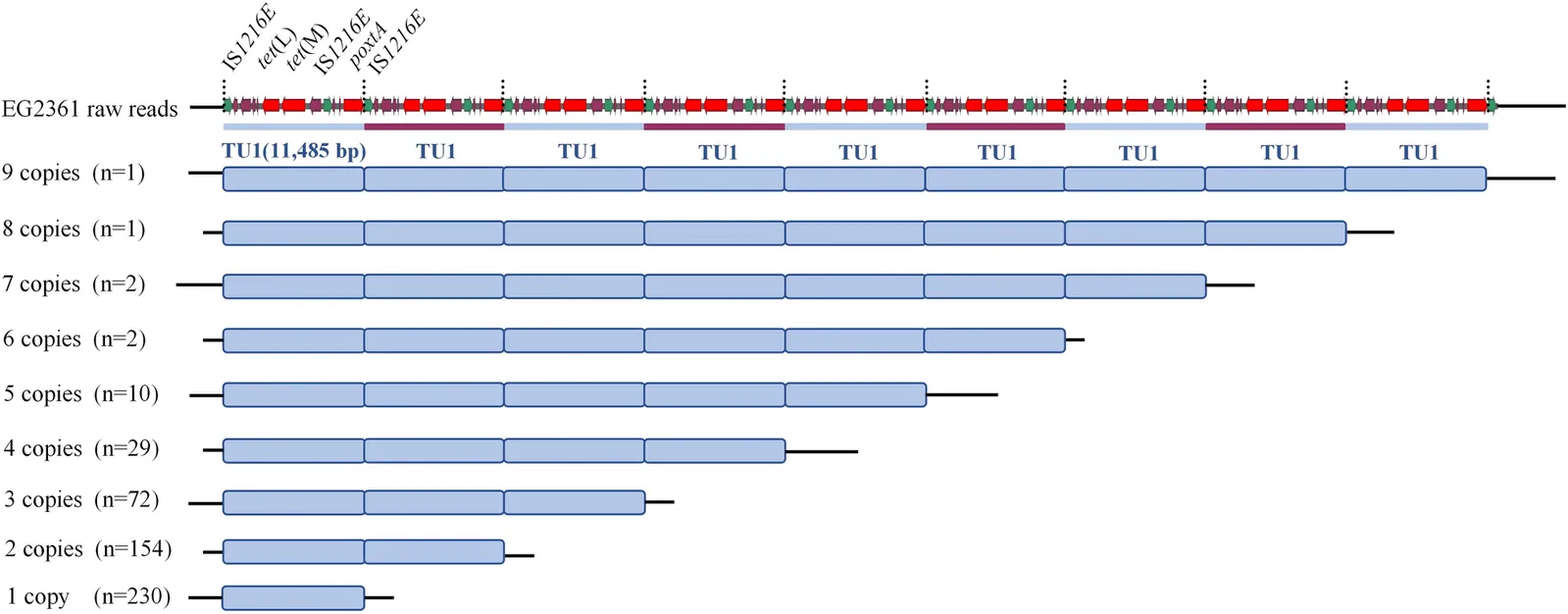
**Tandem repeat variation in MinION reads**. The blue box indicates the repeat unit comprising the IS*1216E–tet*(L)–*tet*(M)–IS*1216E*–*poxtA*–IS*1216E* module. *N*, number of reads containing multiple repeat copies.

To further validate this hypothesis, Oxford Nanopore long-read data were re-analyzed. Reads spanning the repeat region revealed arrays containing one to nine tandem copies of TU1 (Figure 5). Each TU1 unit carried *tet*(L), *tet*(M), *poxtA*, and two IS*1216E* elements in the same orientation. Plasmid size increased proportionally with TU1 copy numbers, with each additional TU1 copy contributing 11,485 bp, consistent with the previous S1-PFGE observations. Although higher-order tandem arrays (e.g., > 10 copies) could not be fully resolved due to read-length limitation, these findings still demonstrate that pEG2361-2 undergoes dynamic structural variation driven by TU1 tandem amplifications, resulting in plasmid heterogeneity through copy number variations.

### Contribution of the promoter variation in *tet*(L)v to tigecycline resistance

Comparative sequence analysis against the reference sequence encoded by plasmid pG38 (GenBank accession no. RCDF01000030) showed that *tet*(L) from the tigecycline-susceptible isolate EG2363 was identical to the reference, whereas *tet*(L) from the tigecycline-resistant isolate EG2361 contained seven amino acid substitutions (Y5F, D43Y, P47T, V94L, V127I, S169Y, and H287Y) and was therefore designated *tet*(L)v (Additional file 2A). In addition, substantial sequence variation was observed in the promoter regions upstream of *tet*(L)v relative to the corresponding region of *tet*(L) (Additional file 2B).

To assess the functional impact of these variations, recombinant *Escherichia coli* DH5α strains carrying *tet*(L) or *tet*(L)v, with or without their respective promoter regions, were constructed. Expression of either *tet*(L) or *tet*(L)v increased MICs to tetracycline (16-fold), doxycycline (4–8-fold), and tigecycline (2-fold) compared with the parental strain (Table 1). However, no significant difference in tigecycline susceptibility was observed between the *tet*(L) and *tet*(L)v constructs, indicating that amino acid substitutions alone do not account for the enhanced resistance phenotype.

**Table 1:** **MICs of antimicrobial agents against the tested strains (mg/L)**

Strain	Abbreviation	Description	Tetracycline	Minocycline	Doxycycline	Tigecycline
DH5α	D5	Recipient strain	1	2	1	0.125
DH5α-pMD19	D5p	Plasmid pMD19 was transferred into the *E.* *coli* DH5α	1	2	1	0.125
DH5α-pMD19_*tet*(L)	D5p*_tet*(L)	DH5α carrying recombinant plasmid pMD19 with the reference *tet*(L) gene	16	2	8	0.25
DH5α-pMD19_*tet*(L) _V_	D5p_*tet*(L) _V_	DH5α carrying recombinant plasmid pMD19 with the *tet*(L) variant gene	16	2	4	0.25
DH5α-pMD19_*tet*(L)_250	D5p*_tet*(L)_250	DH5α carrying recombinant plasmid pMD19 with the reference *tet*(L) gene and its putative promoters	16	2	8	0.25
DH5α-pMD19_*tet*(L)_V__250	D5p*_tet*(L)_V__250	DH5α carrying recombinant plasmid pMD19 with the *tet*(L) variant gene and its putative promoters	32	4	8	0.5

In contrast, inclusion of the promoter region upstream of *tet*(L)v (DH5α-pMD19_*tet*(L)v250) resulted in an additional twofold increase in tigecycline MIC, whereas the construct carrying the wild-type promoter (DH5α-pMD19*tet*(L)_250) showed no detectable changes. These results demonstrate that increased tigecycline resistance is primarily associated with the promoter variations rather than coding-sequence alterations in *tet*(L).

Notably, the *tet*(L)v locus, along with its mutated promoter region, is embedded within TU1, indicating that both the structural gene and its regulatory region are co-amplified during TU1 expansion.

### Amplification of promoter-variant *tet*(L)v drives early tigecycline resistance evolution in *E. hirae* EG2361

**Figure 6:**
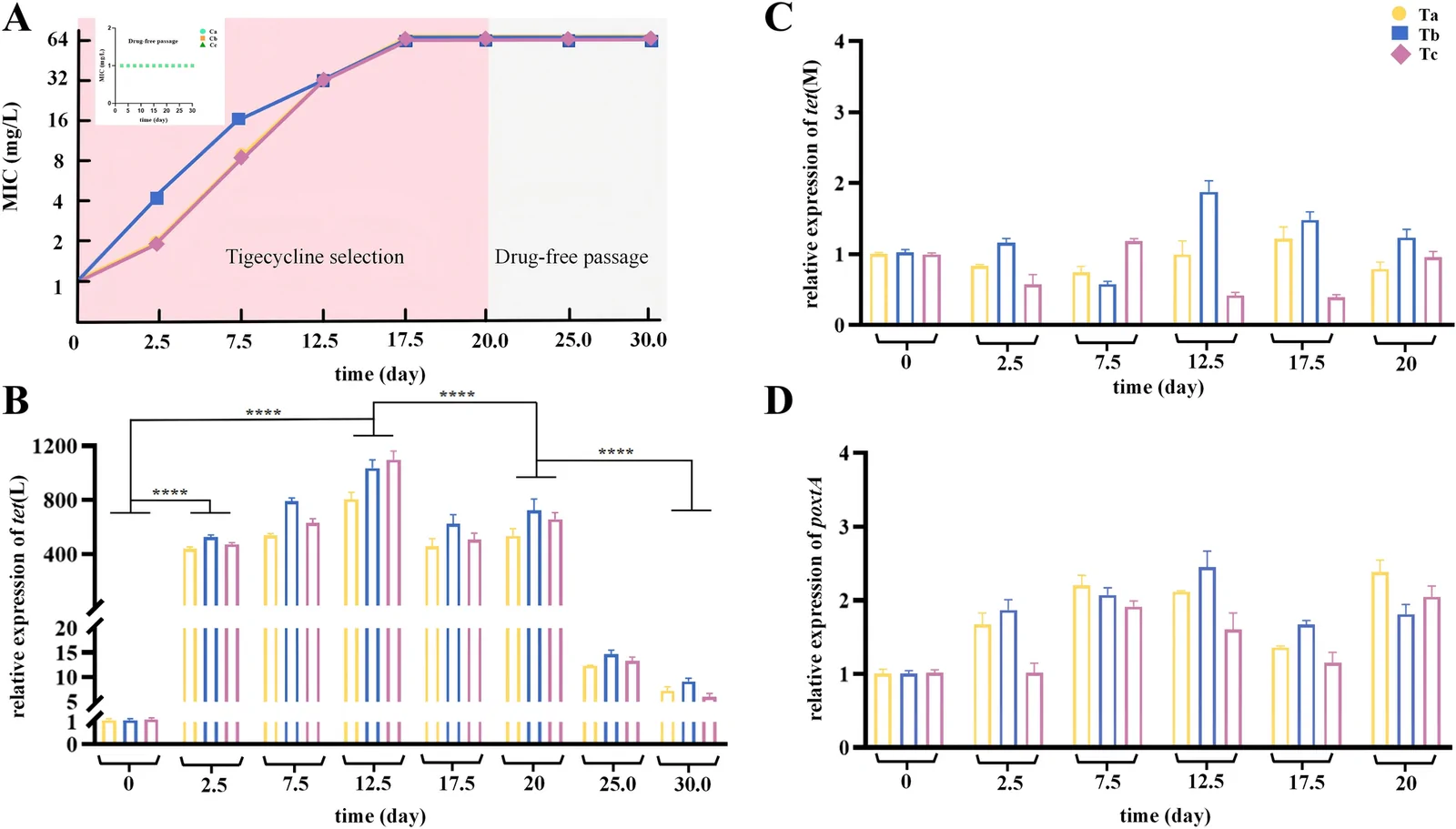
**Tigecycline MICs and gene expression dynamics during experimental evolution of** ***E. hirae*** **EG2361.** **A** Three independent tigecycline-treated lineages (Ta, Tb, and Tc) were passaged under tigecycline selection from day 0 to day 20, followed by drug-free passage from day 25 to day 30. Three control lineages (Ca, Cb, and Cc) were passaged without tigecycline throughout the entire experiment (day 0–30). The light red and light gray backgrounds indicate the tigecycline-selection and drug-free passage phases, respectively. **B** Relative expression levels of *tet*(L) during the 30-day evolution experiment. **C**, **D** Relative expression levels of *tet*(M) and *poxtA* during the tigecycline selection phase (days 0–20).

To investigate the evolution of tigecycline resistance under antibiotic pressure, three independent lineages (Ta, Tb, and Tc) of *E. hirae* EG2361 were serially passaged in the presence of tigecycline, while parallel control lineages (Ca, Cb, and Cc) were passaged without antibiotic selection. Tigecycline MICs of the evolved lineages increased progressively over time and reached 64 mg/L by day 17.5, representing a 64-fold increase relative to the parental strain (Figure 6A). The MICs remained at this level until day 20. After tigecycline withdrawal at day 20, the evolved lineages were passaged for an additional 10 days without antibiotic. Notably, the elevated MICs remained stable at 64 mg/L during days 20–30, indicating that the acquired tigecycline resistance phenotype was maintained even in the absence of selection pressure. In contrast, no MIC changes were observed in the control lineages (Ca, Cb, and Cc) throughout the 30-day passage.

Consistent with the role of TU1 in plasmid heterogeneity, S1-PFGE analysis showed that the size of pEG2361-2 increased markedly during evolution, ranging from approximately 122 kb to more than 244 kb (Additional file 4B), consistent with extensive tandem amplification of TU1. As TU1 carries *tet*(L)v together with its upstream variant promoter region, this amplification resulted in increased copy numbers of both the structural gene and its promoter.

RT-qPCR analysis revealed a dynamic pattern of *tet*(L) expression. In all evolved lineages, *tet*(L) expression levels increased dramatically during the early stage, exceeding 400-fold and peaking at 800–1100-fold on day 12.5 (Figure 6B). Thereafter, its expression levels gradually declined during continued tigecycline exposure, decreasing to approximately 500–750-fold by day 20, and dropped sharply after tigecycline withdrawal to ~5–10-fold above baseline by day 30. In contrast, expression of *tet*(M) and *poxtA* remained largely unchanged throughout the experiment (Figure 6C, D).

It was worth noting that peak *tet*(L) expression preceded the attainment of the highest tigecycline MIC. Specifically, *tet*(L) expression reached its maximum at day 12.5, whereas MICs continued to increase and peaked at 64 mg/L on day 17.5. Although *tet*(L) transcription declined after day 12.5, MICs remained elevated and stable thereafter despite the reduced *tet*(L) expression levels. These results indicate that amplification of the variable promoter region and the resulting overexpression of *tet*(L) drive the early phase of resistance evolution. However, once high-level resistance is established, maintenance of the resistant phenotype no longer appears to depend on sustained high *tet*(L) expression, suggesting a shift toward additional resistance mechanisms during the later stage of evolution.

### Recurrent *rpsJ* mutations are associated with late-stage progression to high-level tigecycline resistance

To investigate whether target-site mutations were associated with the sustained increase in tigecycline MICs following the declined *tet*(L) expression, the *rpsJ* gene encoding ribosomal protein S10 was analyzed. No mutations in *rpsJ* were detected during the early stage of evolution experiment (day 0 to day 7.5) in any of the three independent lineages (Ta, Tb, and Tc) (Table 2), suggesting that the initial increase in tigecycline resistance was primarily associated with amplification of the variable promoter region and elevated *tet*(L) expression levels, rather than *rpsJ* mutations.

**Table 2:** **Emergence of** ***rpsJ*** **mutations during experimental evolution**

Group	Days ^a^	MIC (mg/L)	Amino acid residues of S10 protein (53–60) ^b^								Variant/total
			53	54	55	56	57	58	59	60	
EG2361	0	1	R	A	T	H	K	Y	K	D	/
Ta	2.5	2	R	A	T	H	K	Y	K	D	0/4
	7.5	8	R	A	T	H	K	Y	K	D	0/4
	12.5	32	R	A	T	H	**N**	Y	K	D	3/4
	17.5	64	R	A	T	H	**N**	Y	K	D	4/4
	20	64	R	A	T	H	**N**	Y	K	D	4/4
	25	64	R	A	T	H	**N**	Y	K	D	4/4
	30	64	R	A	T	H	**N**	Y	K	D	4/4
Tb	2.5	2	R	A	T	H	K	Y	K	D	0/4
	7.5	16	R	A	T	H	K	Y	K	D	0/4
	12.5	32	R	A	T	H	**N**	Y	K	D	2/4
	12.5	32	R	**E**	T	H	K	Y	K	D	2/4
	17.5	64	R	A	T	**R**	**N**	Y	K	D	3/4
	20	64	R	A	T	**R**	**N**	Y	K	D	4/4
	25	64	R	A	T	**R**	**N**	Y	K	D	4/4
	30	64	R	A	T	**R**	**N**	Y	K	D	4/4
Tc	2.5	2	R	A	T	H	K	Y	K	D	0/4
	7.5	8	R	A	T	H	K	Y	K	D	0/4
	12.5	32	R	A	T	H	**R**	Y	K	D	2/4
	12.5	32	R	A	T	H	**I**	Y	K	D	2/4
	17.5	64	R	A	T	H	**I**	Y	K	D	4/4
	20	64	R	A	T	H	**I**	Y	K	D	3/4
	25	64	R	A	T	H	**I**	Y	K	D	4/4
	30	64	R	A	T	H	**I**	Y	K	D	4/4

(a) Cultivation was performed under drug selective pressure from days 0 to 20, followed by drug-free cultivation from days 20 to 30. (b) Mutated amino acid residues are indicated in bold.

From day 12.5 onward, nonsynonymous mutations in *rpsJ* emerged in all evolved lineages. Recurrent substitutions were predominantly observed at residue 57, including K57N, K57R, and K57I. In addition, an A54E substitution was detected in one colony of the Tb lineage. At later timepoints (days 17.5 and 20), mutations in the Ta and Tc lineages remained unchanged, whereas the Tb lineage acquired an additional H56R substitution, resulting in double mutations. These mutations persisted during tigecycline-free passages (days 25 and 30), consistent with the stable maintenance of the resistant phenotype.

These findings indicate that *rpsJ* mutations, particularly those affecting residue 57, arose during the later stage of evolution and were closely associated with the progression to high-level tigecycline resistance. Together with the decline in *tet*(L) expression after day 12.5, these results suggest that mechanisms other than *tet*(L) overexpression became increasingly important during the late phase of tigecycline resistance evolution.

## Discussion

Tigecycline remains a critical therapeutic option for infections caused by multidrug-resistant *Enterococcus* spp. [34]. Although tigecycline resistance in enterococci is still relatively uncommon, increasing reports of reduced susceptibility are of growing concern, as they may further compromise treatment efficacy [35]. Food-producing animals are recognized reservoirs of antimicrobial resistance determinants [6], underscoring the importance of investigating tigecycline resistance in animal-associated enterococci from a One Health perspective. In the present study, all tigecycline-resistant *E. hirae* isolates recovered from a swine farm exhibited close relatedness. This finding indicates that clonal expansion may have contributed to the observed tigecycline resistance pattern within this setting, although genome-level comparison of all isolates would be required to confirm their clonal relationships.

Genomic analysis of the representative isolate EG2361 identified a multidrug resistance plasmid, pEG2361-2, carrying *tet*(L), *tet*(M), *poxtA*, *fexB*, and IS*1216E*. The co-localization of multiple resistance genes on a single plasmid may facilitate co-selection under different antimicrobial pressures [36]. As important drivers of bacterial genome rearrangements, insertion sequences (ISs) can facilitate the dissemination of antimicrobial resistance genes (ARGs) in bacteria through transposition, recombination, and the mobilization of resistance-associated DNA segments [37]. Previous studies have primarily reported IS*1216E*-mediated tandem amplification in laboratory-evolved strains, transconjugants, or artificial systems [38, 39]. In contrast, our study demonstrated that such amplification can occur naturally in a resistance plasmid carried by a swine-derived *E. hirae* isolate, revealing intrinsic structural plasticity in the absence of experimental manipulation. Importantly, this preexisting heterogeneity did not confer an obvious resistance phenotype under basal conditions but provided a genetic reservoir for rapid adaptation. Under tigecycline selection, subpopulations with higher TU1 copy numbers were likely preferentially enriched, leading to decreased susceptibility and eventual high-level resistance. This mechanism is consistent with recent evidence that copy-number variation can drive rapid adaptive responses through population heterogeneity [40]. Therefore, tandem amplification in clinical isolates may represent an underappreciated driver of resistance emergence and deserves closer attention.

Functional analyses demonstrated that enhanced tigecycline resistance in EG2361 was primarily associated with variation in the upstream promoter region of *tet*(L)v rather than amino acid substitutions in Tet(L). Although expression of *tet*(L) modestly increased tigecycline MICs, only the construct carrying the variant promoter region produced an additional increase. Similar regulatory mechanisms have been reported for other tigecycline resistance determinants. For example, enhanced promoter activity can upregulate the *tmexCD1-toprJ1* efflux gene cluster [41], while disruption of the *tet*(M) leader peptide can relieve translational attenuation [42], both resulting in increased tigecycline resistance. Together, these findings indicate that regulatory changes, rather than protein structural alterations, can play a central role in shaping tigecycline resistance phenotypes.

Experimental evolution further revealed a stepwise trajectory of tigecycline resistance. During the early phase, tandem amplification of TU1 harboring the promoter-variant *tet*(L)v locus led to marked upregulation of *tet*(L) expression, driving the initial increase in tigecycline MICs. However, amplification-dependent resistance is generally associated with a substantial fitness cost due to increased metabolic burden and genomic instability [43, 44]. As evolution progressed, *tet*(L) expression declined despite the maintenance of high tigecycline MICs, indicating a shift from amplification-dependent resistance toward more stable adaptive strategies.

The structural features of pEG2361-2 support the involvement of IS*1216E* in TU1 tandem amplification. Circular TU1 intermediates were confirmed by inverse PCR and Sanger sequencing, while S1-PFGE, southern blotting, and Nanopore reads revealed stepwise plasmid-size variation and tandem arrays containing multiple TU1 copies. These findings support an IS*1216E*-associated recombination model. Further analysis of repeat junctions and direct comparison of parental and evolved plasmid structures would help clarify the precise amplification mechanism. Although high-level tigecycline resistance remained stable after antibiotic withdrawal, growth, competition, and TU1 copy-number stability assays under tigecycline-free conditions would further define the fitness effects and persistence of this amplification-based resistance mechanism.

Consistent with this transition, mutations in *rpsJ*, which encodes ribosomal protein S10, emerged independently in all evolved lineages during the later stages of tigecycline selection. Mutations at residue 57, in particular, have previously been associated with high-level tigecycline resistance [45]. In the present study, the recurrent emergence of *rpsJ* mutations and their persistence after tigecycline withdrawal suggest that they may contribute to the maintenance of high-level resistance during the later stage of adaptation. Chromosomal *rpsJ* mutations may therefore represent a stable later-stage adaptive route, although their individual functional and fitness effects remain to be experimentally determined. This interpretation is consistent with recent evidence that sustained antibiotic pressure can favor chromosomal mutations that maintain high-level resistance while reducing reliance on potentially costly resistance-gene amplification [46].

Taken together, these findings support a stepwise evolutionary model of tigecycline resistance in *E. hirae*. In the early stage, IS*1216E*-mediated amplification of the promoter-variant *tet*(L)v locus enables rapid adaptive resistance through increased gene dosage and overexpression. During later stages, chromosomal *rpsJ* mutations emerge and become associated with stable high-level resistance, reducing dependence on amplification-driven resistance. In this context, gene amplification appears to function as a rapid-response adaptive mechanism, whereas target-site mutations may represent later-stage adaptations associated with the maintenance of high-level resistance, although their functional effects and fitness consequences remain to be determined.

This study has several limitations. Although cloning experiments demonstrated the functional contribution of the promoter-region variation, the precise promoter activity was not directly quantified using reporter-based assays. The fitness consequences and long-term stability of different TU1 copy-number states under tigecycline-free conditions remain to be further evaluated. In addition, although *rpsJ* mutations repeatedly emerged during independent evolutionary trajectories and were temporally associated with high-level tigecycline resistance, their individual contributions have not been directly validated through genetic reconstruction or mutation-reversion experiments. Furthermore, the transferability of pEG2361-2 and the potential mobilization of the *tet*(L)v-containing TU1 to other enterococcal species was not experimentally evaluated and warrants further investigation. Finally, broader epidemiological investigations are needed to assess the prevalence of IS*1216E*-associated resistance plasmids and amplification events in livestock-associated enterococci.

## Conclusions

This study reveals a stepwise evolutionary pathway of tigecycline resistance in *E. hirae*, involving IS*1216E*-mediated tandem amplification of the promoter-variant *tet*(L)v locus followed by the emergence of chromosomal *rpsJ* mutations associated with stable high-level resistance. These findings highlight the interplay among mobile genetic elements, transcriptional regulation, and chromosomal adaptation, and emphasize the importance of genome structural plasticity in driving antimicrobial resistance evolution in both clinical and agricultural settings.

## Supplementary Information

**Additional file 1. Primers used in this study.**

**Additional file 2.** **Sequence analysis of** ***tet*(L) and its upstream promoter region.**

**Additional file 3.** **Species distribution and antimicrobial resistance rates of** ***Enterococcus*** **spp. isolates.**

**Additional file 4.** **PFGE profiles of tigecycline-resistant** ***E. hirae*** **isolates and S1-PFGE analysis of** ***E. hirae*** **EG2361 during experimental evolution.**

**Additional file 5.** **Genomic features of** ***E. hirae*** **EG2361.**

**Additional file 6. Identification of TUs in plasmid pEG2361-2.**

**Additional file 7.** **S1-PFGE plasmid profiles of** ***E. hirae*** **EG2361 single colonies.**

## Data Availability

No datasets were generated or analyzed during the current study.
