# Supplementary material for: Stepwise evolution of tigecycline resistance in Enterococcus hirae through early IS1216E-mediated tet(L) amplification and subsequent rpsJ mutations
Source: Vet Res. 2026 Oct 10;57:179. doi: 10.1186/s13567-026-01853-4 (PMC13654235; doi:10.1186/s13567-026-01853-4)
Supplement: Supplementary file 1 [file 13567_2026_1853_MOESM1_ESM.docx]

**Additional file 1**. Primers used in this study.

| Name | Primer sequences（5′-3′） | Product size (bp) | Reference |
| --- | --- | --- | --- |
| **Primers used for TU identification** | | | |
| P1/P2 products | P1: ACACGAGTATCCATGAATCTAC  P2: AATGGGTGGCAATTATTAGACC | 13,476/5,312/1,991 | This study |
| TU1 | P3: AGTCGAGATCAGGGAATGAG  P4: AAGCCAGTCAGACCAATCATCA | 2,183 | This study |
| TU2 | P5: GTGTAGAGCCAAAATCAATGC  P6: CCGTACTGAGCAACTTCCTGTT | 1,656 | This study |
| TU3 | P3: AGTCGAGATCAGGGAATGAG  P7: GTACCCACAAGGCAATCAC | 1,696 | This study |
| **Primers used for the cloning experiment** | | | |
| *tet*(L) | F: GCCCTCATTATTGGAGGGTGAAAT  R: TCCCAAAGTTGATCCCTTAACGAT | 1,425 | This study |
| *tet*(L)v | F: GCCCTCATTATTGGAGGTTGAAAT  R: TCCCAAAGTTGATCCCTTAACGAT | 1,425 | This study |
| *tet*(L)_250 | F: AGTATTAGTAATTATCATGGCTGT  R: TCCCAAAGTTGATCCCTTAACGAT | 1,651 | This study |
| *tet*(L)v_250 | F: CGTCGTCCAAATAGTCGGATAGAT  R: TCCCAAAGTTGATCCCTTAACGAT | 1,651 | This study |
| **Primers used for RT-qPCR** | | | |
| 16S rDNA | F: GCAAGGTTGAAACTCAAAGG  R: CTAGAGTGGTCAAAGGATGTC | 116 | This study |
| *tet*(L) | F: TCTTATCGTTAGCGTGCTGTCA  R: TTTCCCTAATCCGGGATCAACA | 88 | This study |
| *tet*(M) | F: ACAACGAGGACGGATAATAC  R: CGTGTCTATGATGTTCACCT | 134 | This study |
| *poxtA* | F: CCGTACTGAGCAACTTCCTGTT  R: GGTCGGCTCGTCCAGTATCAA | 140 | This study |
| **Primers for *rpsJ* gene sequencing** | | | |
| *rpsJ* | F: TCGTGGAGCTATGTCTACTT  R: CCTAAGATTCCTTTGGTCAT | 406 | This study |

Note: P1/P2 amplicons were classified into three types: containing TU1 only, containing TU2 only, or containing neither TU1 nor TU2. No amplification was obtained with primer pair P3/P7.
