# Supplementary material for: Stepwise evolution of tigecycline resistance in Enterococcus hirae through early IS1216E-mediated tet(L) amplification and subsequent rpsJ mutations
Source: Vet Res. 2026 Oct 10;57:179. doi: 10.1186/s13567-026-01853-4 (PMC13654235; doi:10.1186/s13567-026-01853-4)
Supplement: Supplementary file 2 [file 13567_2026_1853_MOESM2_ESM.docx]

**Additional file 2.** Sequence analysis of *tet*(L) and its upstream promoter region. (A) Amino acid sequence alignment of Tet(L) from the tigecycline-resistant isolate *E. hirae* EG2361 and the tigecycline-susceptible isolate EG2363, together with the reference Tet(L) sequence encoded by plasmid pG38 (GenBank accession no. RCDF01000030). (B) Alignment of the 250-bp upstream region of *tet*(L) from EG2361 with the corresponding reference sequence, showing sequence variation in the predicted promoter region. Lowercase letters indicate nucleotides that differ from the reference sequence, hyphens indicate nucleotides identical to the reference sequence, and dots represent gaps introduced during sequence alignment. Red arrows indicate the primer-binding sites used for cloning.
