# Supplementary material for: Stepwise evolution of tigecycline resistance in Enterococcus hirae through early IS1216E-mediated tet(L) amplification and subsequent rpsJ mutations
Source: Vet Res. 2026 Oct 10;57:179. doi: 10.1186/s13567-026-01853-4 (PMC13654235; doi:10.1186/s13567-026-01853-4)
Supplement: Supplementary file 3 [file 13567_2026_1853_MOESM3_ESM.docx]

**Additional file 3**. Species distribution and antimicrobial resistance rates of *Enterococcus* spp. isolates

| Species | Rectal swabs | Pharyngeal  swabs | Totals | Isolation rates (%) | Resistance rates of antimicrobial agents (%) | | | | | | | |
| --- | --- | --- | --- | --- | --- | --- | --- | --- | --- | --- | --- | --- |
|  |  |  |  |  | LZD | FFC | VAN | DOX | TGC | EN | TYL | LIN |
| *E. hirae* | 67 | 1 | 68 | 57.63 | 0 | 100 (68/68) | 0 | 100 (68/68) | 14.71 (10/68) | 94.12 (64/68) | 98.53 (67/68) | 100 (68/68) |
| *E. faecalis* | 3 | 0 | 3 | 2.54 | 0 | 100 (3/3) | 0 | 100 (3/3) | 0 | 66.67 (2/3) | 100 (3/3) | 100 (3/3) |
| *E. faecium* | 2 | 16 | 18 | 15.25 | 0 | 55.56 (10/18) | 0 | 22.22 (4/18) | 0 | 22.22 (4/18) | 16.67 (3/18) | 38.89 (7/18) |
| *E. durans* | 1 | 13 | 14 | 11.86 | 0 | 35.71 (5/14) | 0 | 14.29 (2/14) | 0 | 14.29 (2/14) | 0 | 14.29 (2/14) |
| *E.* *casseliflavus* | 0 | 11 | 11 | 9.32 | 0 | 27.27 (3/11) | 0 | 0 | 0 | 9.09 (1/11) | 0 | 54.55 (6/11) |
| *E. mundtii* | 0 | 4 | 4 | 3.39 | 0 | 100 (4/4) | 0 | 25.00 (1/4) | 0 | 25.00 (1/4) | 0 | 100 (4/4) |
| Subtotal | 73 | 45 | 118 | 100 | 0 | 78.81 (93/118) | 0 | 66.10 (78/118) | 8.47 (10/118) | 62.71 (74/118) | 61.86 (73/118) | 76.27 (90/118) |

Note: LZD, linezolid, FFC, florfenicol, VAN, vancomycin, DOX, doxycycline, TGC, tigecycline, EN, enrofloxacin, TYL, tylosin, LIN, lincomycin.
