# Supplementary material for: Stepwise evolution of tigecycline resistance in Enterococcus hirae through early IS1216E-mediated tet(L) amplification and subsequent rpsJ mutations
Source: Vet Res. 2026 Oct 10;57:179. doi: 10.1186/s13567-026-01853-4 (PMC13654235; doi:10.1186/s13567-026-01853-4)
Supplement: Supplementary file 4 [file 13567_2026_1853_MOESM4_ESM.docx]

**Additional file 4.** PFGE profiles of tigecycline-resistant *E. hirae* isolates and S1-PFGE analysis of *E. hirae* EG2361 during experimental evolution. (A) Genomic DNA from ten isolates was digested with *Sma*I and analyzed by PFGE. Lanes 1–10: tigecycline-resistant *E. hirae* isolates. (B) Lanes 1–5 represent the Ta lineage at days 2.5, 7.5, 12.5, 17.5, and 20; lanes 6–10, Tb lineage; lanes 11–15, Tc lineage at the same time points. Lane 16 shows the strain passaged for 20 days under tigecycline-free conditions. M, *Salmonella enterica* serotype Braenderup H9812.
