# Supplementary material for: Stepwise evolution of tigecycline resistance in Enterococcus hirae through early IS1216E-mediated tet(L) amplification and subsequent rpsJ mutations
Source: Vet Res. 2026 Oct 10;57:179. doi: 10.1186/s13567-026-01853-4 (PMC13654235; doi:10.1186/s13567-026-01853-4)
Supplement: Supplementary file 5 [file 13567_2026_1853_MOESM5_ESM.docx]

**Additional file 5**. Genomic features of *E. hirae* EG2361

| Sequences | Lengths (bp) | GC contents（%） | Drug resistance genes | Insert sequences | Plasmid replicons |
| --- | --- | --- | --- | --- | --- |
| Chromosome | 2, 920, 071 | 36.89 | *aac*(6')*-Iid* | / | */* |
| pEG2361-1 | 137, 737 | 35.53 | *aph*(3')*-IIIa*, *ant*(6)*-Ia*, *ant*(9)*-Ia*, *lnu*(B), *lsa*(E), *erm*(B) | IS*Efm1*, IS*Ef1*  IS*1216E*, IS*1485*, IS*1182*, IS*1297* | repUS15 |
| pEG2361-2 | 33, 213 | 35.75 | *poxtA*, *tet*(L), *tet*(M), *fexB* | IS*1216E*, IS*1252* | rep2 |
| pEG2361-3 | 23, 624 | 34.40 | *erm*(B), *dfrG* | IS*Ef1*, IS*1542*, IS*1216E* | repUS1 |
