# Supplementary material for: Stepwise evolution of tigecycline resistance in Enterococcus hirae through early IS1216E-mediated tet(L) amplification and subsequent rpsJ mutations
Source: Vet Res. 2026 Oct 10;57:179. doi: 10.1186/s13567-026-01853-4 (PMC13654235; doi:10.1186/s13567-026-01853-4)
Supplement: Supplementary file 6 [file 13567_2026_1853_MOESM6_ESM.docx]

**Additional file 6.** Identification of TUs in plasmid pEG2361-2. (A) PCR products obtained with primers P1/P2 (~13,476 bp, ~5,312 bp, and ~1,991 bp). (B) PCR product obtained with primers P3/P4 (~2,183 bp). (C) PCR product obtained with primers P5/P6 (~1,656 bp). M, DNA marker; N, negative control.
