# Supplementary material for: Stepwise evolution of tigecycline resistance in Enterococcus hirae through early IS1216E-mediated tet(L) amplification and subsequent rpsJ mutations
Source: Vet Res. 2026 Oct 10;57:179. doi: 10.1186/s13567-026-01853-4 (PMC13654235; doi:10.1186/s13567-026-01853-4)
Supplement: Supplementary file 7 [file 13567_2026_1853_MOESM7_ESM.docx]

**Additional file 7**. S1-PFGE plasmid profiles of *E. hirae* EG2361 single colonies

| S1-PFGE types | Plasmids (kb) | | pEG2361-2 + TU1 copies  (kb) | Single colony of EG2361 | Percentages (%) |
| --- | --- | --- | --- | --- | --- |
|  | pEG2361-1 | pEG2361-2 |  |  |  |
| Type Ⅰ | ≈138 | ≈155 | 33+11× TU1 | S5 | 10 |
| Type Ⅱ | ≈138 | ≈133 | 33+9× TU1 | S1、S6、S7 | 30 |
| Type Ⅲ | ≈138 | ≈89 | 33+5× TU1 | S2 | 10 |
| Type Ⅳ | ≈138 | ≈67 | 33+3× TU1 | S3、S10 | 20 |
| Type Ⅴ | ≈138 | ≈56 | 33+2× TU1 | S9 | 10 |
| Type Ⅵ | ≈138 | ≈45 | 33+1×TU1 | S4、S8 | 20 |

Note: Small plasmids (pEG2361-3) may not be clearly resolved by S1-PFGE under the conditions used.
